# Author Correction: The scaffold protein p140Cap limits ERBB2-mediated breast cancer progression interfering with Rac GTPase-controlled circuitries

**DOI:** 10.1038/ncomms16203

**Published:** 2018-03-30

**Authors:** Silvia Grasso, Jennifer Chapelle, Vincenzo Salemme, Simona Aramu, Isabella Russo, Nicoletta Vitale, Ludovica Verdun di Cantogno, Katiuscia Dallaglio, Isabella Castellano, Augusto Amici, Giorgia Centonze, Nanaocha Sharma, Serena Lunardi, Sara Cabodi, Federica Cavallo, Alessia Lamolinara, Lorenzo Stramucci, Enrico Moiso, Paolo Provero, Adriana Albini, Anna Sapino, Johan Staaf, Pier Paolo Di Fiore, Giovanni Bertalot, Salvatore Pece, Daniela Tosoni, Stefano Confalonieri, Manuela Iezzi, Paola Di Stefano, Emilia Turco, Paola Defilippi

Nature Communications 8: Article number: 14797; DOI: 10.1038/ncomms14797 (2017); Published online 03 16 2017; Updated 03 30 2018

In the original version of this Article, the affiliation details for Anna Sapino were incorrectly given as Department of Medical Sciences, University of Torino, 10126 Torino, Italy instead of Candiolo Cancer Institute-FPO, IRCCS, Str. Prov. 142, km 3.95, I-10060, Candiolo (To), Italy. This has now been corrected in both the PDF and HTML versions of the Article.

