# Supplementary material for: The scaffold protein p140Cap limits ERBB2-mediated breast cancer progression interfering with Rac GTPase-controlled circuitries
Source: Nat Commun. 2017 Mar 16;8:14797. doi: 10.1038/ncomms14797 (PMC5357316; doi:10.1038/ncomms14797)
Supplement: Supplementary Information — Supplementary Figures, Supplementary Tables and Supplementary Methods [file ncomms14797-s1.pdf]

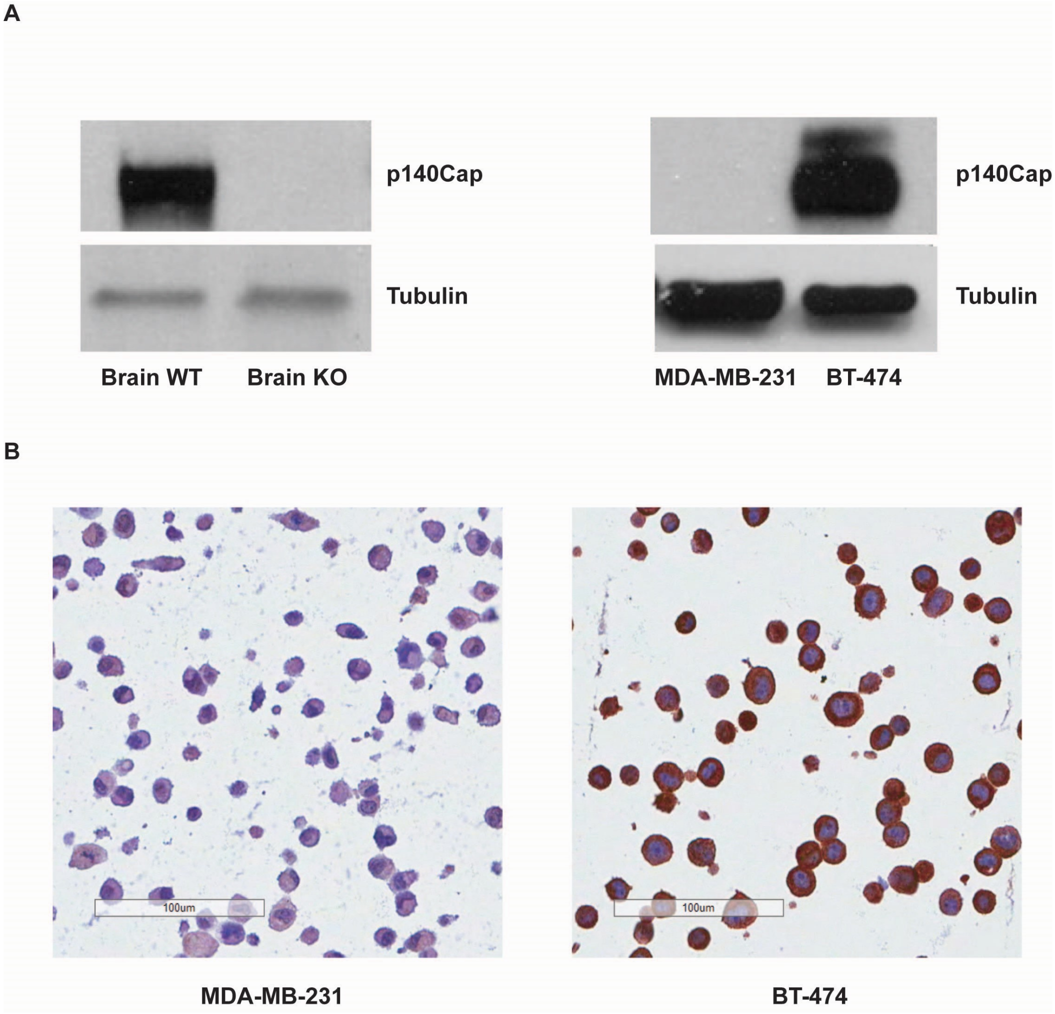

Supplementary Figure 1: Characterization of p140Cap monoclonal antibodies.

(A) The purified monoclonal antibody generated as described in Supplementary Methods, was tested in western blotting for specificity on 10 micrograms of brain tissue extract from WT and p140Cap KO mice. As expected, WT brain extracts show a clear band of 140 kDal MW, while KO brain extracts were negative, even at long exposure. Further, the antibody was tested for reactivity on human cells, in western blot of 40 micrograms of human breast cancer cell extracts (MDA-MB-231 and BT-474). As shown, BT-474 cells were highly positive, while MDA-MB-231 were negative.

(B) The same antibody was tested for IHC, as reported in Methods, on formalin-fixed paraffin-embedded pellets of MDA-MB-231 (left, chosen as negative cells) and BT-474 (right, chosen as positive control cells). Bars are 100 microns.

A

All Patients, Distant Relapse Free Interval

Univariate

	Level1	/Level2	Risk Ratio	P value	Lower 95%	Upper 95%
**p140**	**HIGH**	**LOW**	**0.5720149**	**0.0365**	0.350505	0.9641135

Multivariate

	Level1	/Level2	Risk Ratio	P value	Lower 95%	Upper 95%
p140	HIGH	LOW	0.8706766	0.6133	0.5169868	1.5091233
**pT**	**pT1**	**pT2-pT4**	**0.448407**	**0.0027**	0.2628374	0.7572243
**LN Status**	**NEG**	**POS**	**0.4575175**	**0.0035**	0.2587361	0.7778191
**Grade**	**G1-G2**	**G3**	**0.4198032**	**0.0024**	0.2342872	0.7362633
ER/PgR	NEG	POS	0.5468677	0.0947	0.2489433	1.1052836
Ki67	<14%	>=14%	0.6112612	0.1811	0.2824583	1.2519491
ERBB2	NEG	POS	0.9703775	0.9211	0.5483092	1.8094748

All Patients, Death Related to Breast Cancer

Univariate

	Level1	/Level2	Risk Ratio	P value	Lower 95%	Upper 95%
**p140**	**HIGH**	**LOW**	**0.5319576**	**0.02**	0.3233286	0.9017938

Multivariate

	Level1	/Level2	Risk Ratio	P value	Lower 95%	Upper 95%
p140	HIGH	LOW	0.8513093	0.5593	0.5032618	1.4786391
**pT**	**pT1**	**pT2-pT4**	**0.4209466**	**0.0018**	0.2394586	0.7265603
**LN Status**	**NEG**	**POS**	**0.5685381**	**0.0375**	0.3229235	0.9684396
**Grade**	**G1-G2**	**G3**	**0.4314931**	**0.0056**	0.2312214	0.7833843
ER/PgR	NEG	POS	0.8377956	0.5991	0.4215875	1.605592
Ki67	<14%	>=14%	0.5735595	0.1828	0.2335321	1.2894557
ERBB2	NEG	POS	0.7314684	0.2955	0.4160667	1.3256922

ERBB2 Negative, Distant Relapse Free Interval

Univariate

p140	Level1	/Level2	Risk Ratio	P value	Lower 95%	Upper 95%
	HIGH	LOW	0.7420398	0.3474	0.4148845	1.4103109

Multivariate

	Level1	/Level2	Risk Ratio	P value	Lower 95%	Upper 95%
p140	HIGH	LOW	1.0831685	0.809	0.5783348	2.1454341
**pT**	**pT1**	**pT2-pT4**	**0.4081718**	**0.0037**	0.2209329	0.7474067
LN Status	NEG	POS	0.5683	0.0681	0.2978487	1.0420703
**Grade**	**G1-G2**	**G3**	**0.4026185**	**0.0041**	0.2133278	0.7486085
**ER/PgR**	**NEG**	**POS**	**0.334771**	**0.0467**	0.078222	0.9857983
Ki67	<14%	>=14%	0.4774126	0.0604	0.2048179	1.0321826

ERBB2 Negative, Death Related to Breast Cancer

Univariate

	Level1	/Level2	Risk Ratio	P value	Lower 95%	Upper 95%
p140	HIGH	LOW	0.7953589	0.5164	0.4176017	1.6421369

Multivariate

	Level1	/Level2	Risk Ratio	P value	Lower 95%	Upper 95%
p140	HIGH	LOW	1.3031398	0.4694	0.6482397	2.8285933
**pT**	**pT1**	**pT2-pT4**	**0.3189052**	**0.0008**	0.1583263	0.6252334
LN Status	NEG	POS	0.8755408	0.7005	0.4346963	1.708836
**Grade**	**G1-G2**	**G3**	**0.3365972**	**0.0016**	0.1652133	0.6631855
ER/PgR	NEG	POS	0.4471501	0.16	0.1040201	1.328516
Ki67	<14%	>=14%	0.4512981	0.092	0.1588231	1.1341458

ERBB2 Positive, Distant Relapse Free Interval

Univariate

	Level1	/Level2	Risk Ratio	P value	Lower 95%	Upper 95%
**p140**	**HIGH**	**LOW**	**0.2967936**	**0.0186**	0.1088141	0.8094659

Multivariate

	Level1	/Level2	Risk Ratio	P value	Lower 95%	Upper 95%
p140	HIGH	LOW	0.4549375	0.1483	0.1524208	1.3305152
pT	pT1	pT2-pT4	0.6367873	0.4456	0.18387	2.0170165
**LN Status**	**NEG**	**POS**	**0.2667265**	**0.0247**	0.0599776	0.854786
Grade	G1-G2	G3	0.3744074	0.1762	0.0561657	1.4914255
ER/PgR	NEG	POS	1.1448957	0.8117	0.3702801	3.575178
Ki67	<14%	>=14%	3.9444687	0.1919	0.4608243	33.916178

ERBB2 Positive, Death Related to Breast Cancer

Univariate

	Level1	/Level2	Risk Ratio	P value	Lower 95%	Upper 95%
**p140**	**HIGH**	**LOW**	**0.2932358**	**0.006**	0.1219635	0.696787

Multivariate

	Level1	/Level2	Risk Ratio	P value	Lower 95%	Upper 95%
**p140**	**HIGH**	**LOW**	**0.3555117**	**0.0304**	0.1360506	0.9056813
pT	pT1	pT2-pT4	0.8522348	0.762	0.2903289	2.3850379
**LN Status**	**NEG**	**POS**	**0.3456328**	**0.0328**	0.1098542	0.9199781
Grade	G1-G2	G3	0.7678905	0.6648	0.2020121	2.426359
ER/PgR	NEG	POS	2.1473093	0.1373	0.7872955	6.3634635
Ki67	<14%	>=14%	2.8766994	0.2686	0.3833861	15.766078

B

ERBB2 Positive, Distant Relapse Free Interval

Multivariate

	Level1	/Level2	Risk Ratio	P value	Lower 95%	Upper 95%
pT	pT1	pT2-pT4	0.7162612	0.478	0.2717615	1.7893757
**LN Status**	**NEG**	**POS**	**0.2337018**	**0.0042**	0.0658566	0.650615
Grade	G1-G2	G3	0.4127382	0.1372	0.0938687	1.2951061
ER/PgR	NEG	POS	1.4288026	0.4774	0.5281006	3.8741004
Ki67	<14%	>=14%	3.8453004	0.1211	0.679826	21.904925

Bivariate

	Level1	/Level2	Risk Ratio	P value	Lower 95%	Upper 95%
**p140**	**HIGH**	**LOW**	**0.3344107**	**0.0338**	0.1220123	0.9163062
**LN Status**	**NEG**	**POS**	**0.2759502**	**0.0254**	0.0629726	0.8633479

ERBB2 Positive, Death Related to Breast Cancer

Multivariate

	Level1	/Level2	Risk Ratio	P value	Lower 95%	Upper 95%
pT	pT1	pT2-pT4	0.8166642	0.6369	0.3408016	1.8819983
**LN Status**	**NEG**	**POS**	**0.2979287**	**0.0066**	0.1063222	0.7237146
Grade	G1-G2	G3	0.7255563	0.5399	0.2292479	1.9494093
ER/PgR	NEG	POS	2.0636492	0.1156	0.8373206	5.2968011
Ki67	<14%	>=14%	2.773088	0.204	0.5394967	12.209608

Bivariate

	Level1	/Level2	Risk Ratio	P value	Lower 95%	Upper 95%
**p140**	**HIGH**	**LOW**	**0.3186571**	**0.0105**	0.1321398	0.7595067
**LN Status**	**NEG**	**POS**	**0.3571535**	**0.0317**	0.1162745	0.9178043

Supp Fig. 2

Supplementary Figure 2. Cox proportional regression analysis of p140Cap expression in Breast Cancer.

(A) Left panels: Distant Relapse Free Interval univariate and multivariate analysis of p140Cap expression in the whole cohort of breast cancer patients (upper tables), in ERBB2-negative patients (middle tables) and in ERBB2-positive patients (lower tables). Right Panels: Survival to Death Related to Breast Cancer univariate and multivariate analysis of p140Cap expression in the whole cohort of breast cancer patients (upper panels), in ERBB2-negative patients (middle panels) and in ERBB2-positive patients (lower panels).

(B) Left panels: Distant Relapse Free Interval multivariate analysis of standard clinical-pathological parameter (upper table) and bivariate analysis with p140Cap and Lymph-node status (lower table) in ERBB2-Positive patients. Right panels: Survival to Death Related to Breast Cancer multivariate analysis of standard clinical-pathological parameter (upper table) and bivariate analysis with p140Cap and Lymph-node status (lower table) in ERBB2-Positive patients.

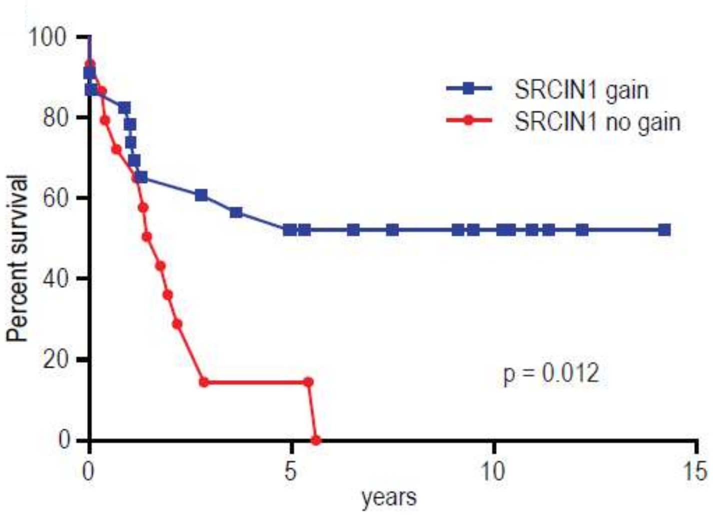

Supplementary Figure 3. Kaplan-Meier survival curves based on *SRCIN1* copy number variations on *ERBB2* amplified patients.

Survival percentage of patients with *SRCIN1* gain (blue line) was compared to that of patients with *SRCIN1* no gain (red line). Y-axis corresponds to the percentage of survival. p=0.012. The patients cohort was the same showed in Figure 2A.

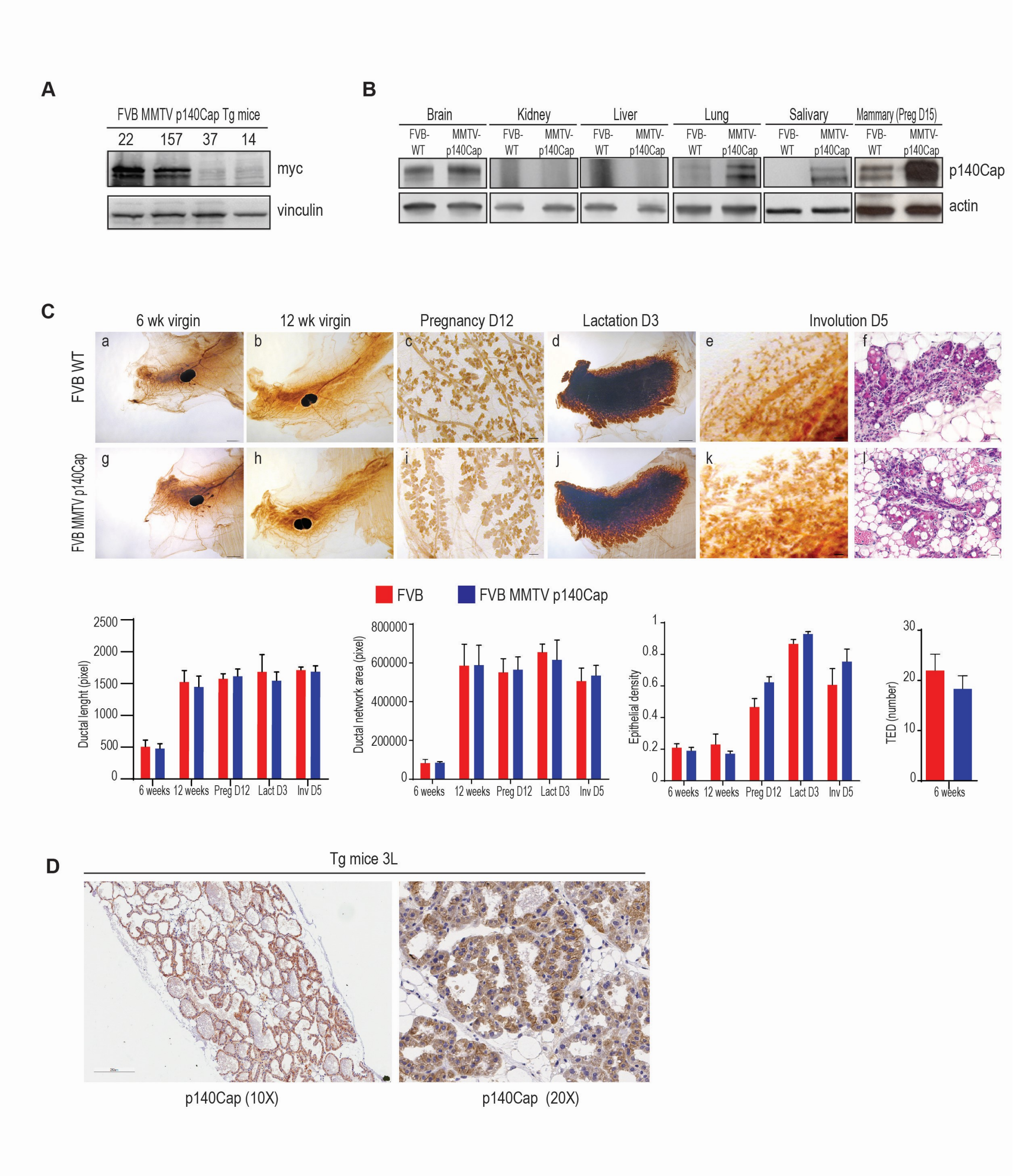

Supplementary Figure 4. Characterization of MMTV-p140Cap transgenic mice

(A) p140Cap expression in MMTV-p140Cap transgenic mouse lines. Mammary gland tissue protein extracts were prepared at three days lactation from different Tg female mice (22, 157, 37 and 14), scored positive by PCR and Southern blot. Two of them (22 and 157) showed high and comparable p140Cap expression in the mammary gland. Protein extracts run on 6% SDS-PAGE were stained with antibodies to Myc directed to the transgene p140Cap-Myc (upper panel) and vinculin for loading control (lower panel).

(B) p140Cap expression in different tissues of the MMTV-p140Cap transgenic mice. Protein extracts from distinct tissues collected from FVB WT or MMTV-p140Cap Tg female at 15 days of pregnancy, were run on 6% SDS-PAGE and stained with antibodies to p140Cap or to actin, as loading control. p140Cap transgene is expressed in mammary glands, parotid glands, lungs, and, at a minor extent, in brain. Representative images out of four different animals are shown.

(C) p140Cap expression in Tg mice does not impair mammary gland development and differentiation. Mammary glands from WT and MMTV-p140Cap transgenic mice were collected at several stages of physiologic mammary development, namely at 6 weeks (puberal period) and 12 weeks (mature virgin mammary gland) of age, 12 days of pregnancy, 3 days of lactation and 5 days of post weaning involution. Five parameters were analysed to compare WT and Tg mammary glands (see below), and reported in histograms (mean ± SEM). No differences in TEB number, ductal elongation and ductal network area during mammary gland growth and development in virgin mice, a non significant trend to lobular increase during pregnancy and lactation and a small impairment in post weaning lobular involution in Tg mice were observed. Bar: a, b, d, g, h, j 0,2 cm; c, e, i, k 300 µm; f, l 20 µm.

(D) p140Cap is expressed into the mammary epithelial compartment of the Tg mice. Formalin-fixed paraffin-embedded fourth abdominal mammary glands of Tg mice at 3 days of lactation were analyzed by IHC with anti p140Cap antibodies, as shown in Figure 1. The staining reveals a clear epithelial localization of p140Cap.

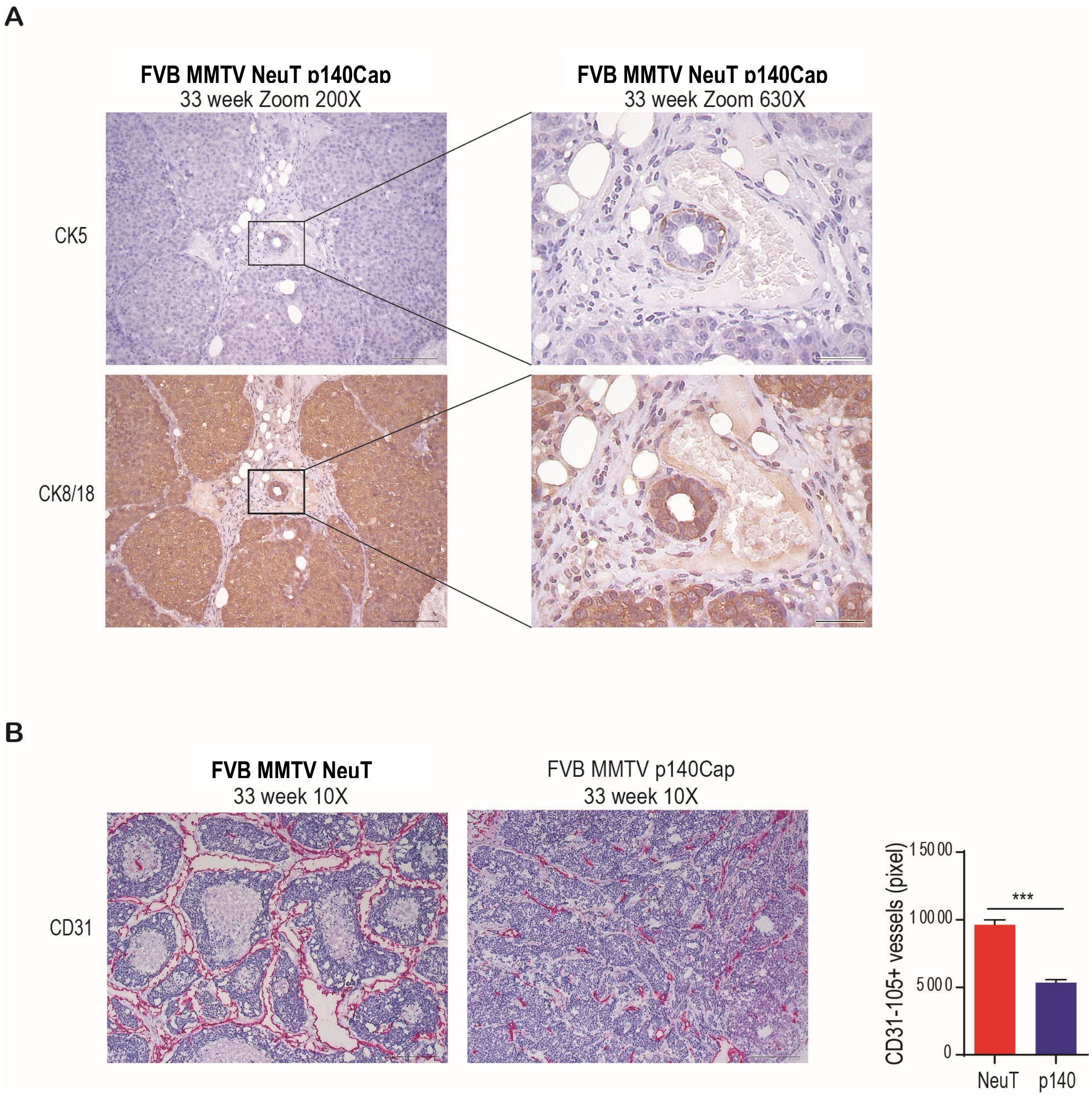

Supplementary Figure 5. Characterization of luminal status and angiogenic infiltration in NeuT and p140 tumors

(A) Formalin fixed paraffin embedded sections from three NeuT and three p140-NeuT tumors were analyzed for immunohystochemistry with antibodies to CK5 and CK8-18. Figure shows representative images of a p140-NeuT tumors in which a normal residual duct is outlined by a continuous layer of CK5 positive basal cells while the whole surrounding tumor is completely negative. On the contrary, both luminal duct cells and the whole tumor are positive for the CK8-18

staining indicating that p140 expression does not alter the luminal status of the tumor. Bar x,x 200 microns. Bar 30 microns.

(B) Cryo-sections from three NeuT and three p140-NeuT tumors taken from mice at 33 weeks of age were analyzed for immunohystochemistry with antibodies against endothelial cells: CD31 and CD105. Representative images are shown. Bar 200 microns. Histogram indicates the number of red pixels counted in 6 low power fields per tumor. Scale bar 200 microns.

A

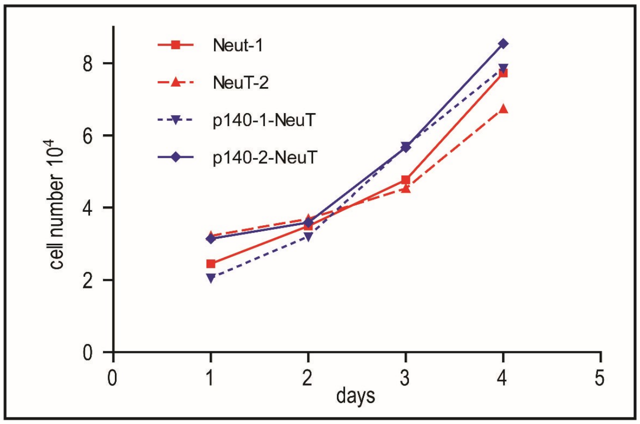

B

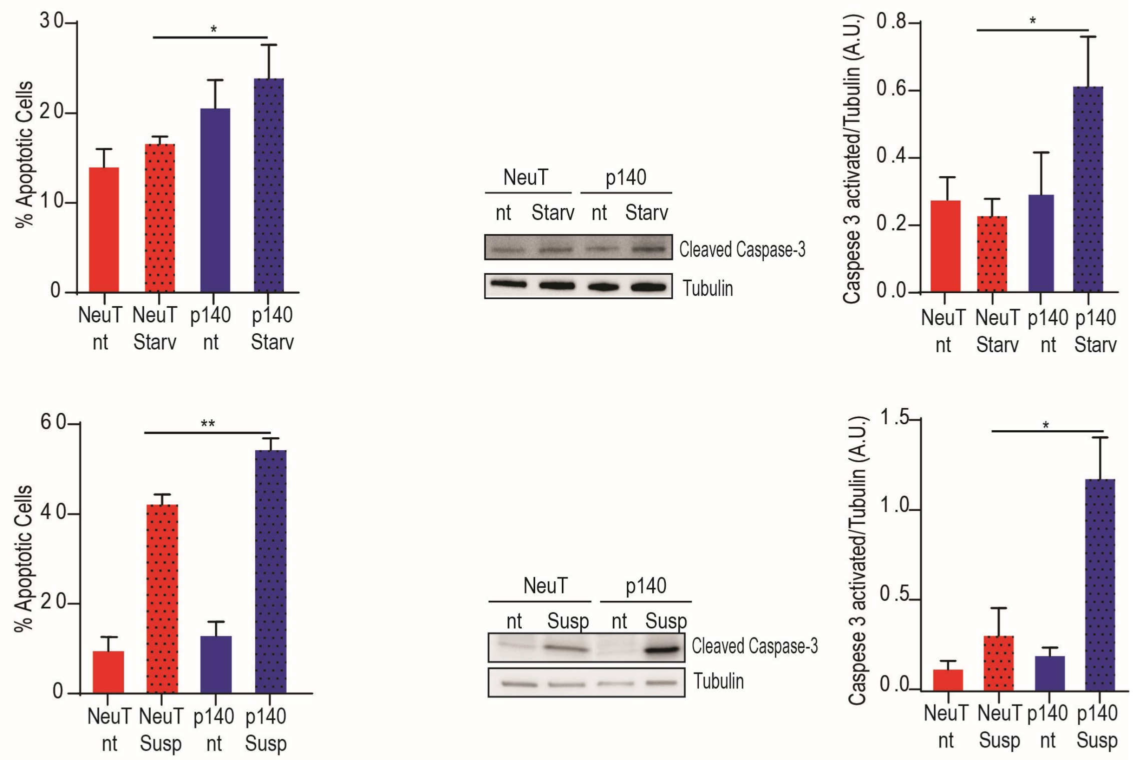

Supplementary Figure 6. Cell proliferation and survival ability of NeuT/p140 primary cancer cells

(A) Cell proliferation analysis of NeuT/p140 primary cancer cells. 2 x 10^4 NeuT-1, NeuT-2, p140-1-NeuT and p140-2-NeuT cells were seeded on 12 well tissue culture dishes in the presence of the culture medium. Cells were detached and manually counted in Burker chambers on triplicate wells every day. These experiments were performed in triplicate.

(B) p140Cap sensitizes the cells to apoptotic stimuli.

Left panels. The percentage of apoptotic cells were measured by FACS analysis (Annexin V and propidium iodide staining) in NeuT or Mock SKBR3 cells (red bars), and p140-NeuT and p140 over-expressing SKBR3 cells (blu bars). Cells were subjected to 12 hours of starvation, or kept in suspension for 12.

Middle panels. Protein extracts from NeuT/p140 cells and Mock/p140 SKBR3 cells treated as above, were probed with antibodies to Cleaved Caspase 3 and Tubulin as loading control.

Right panels. Quantification of Cleaved Caspase 3 expression in arbitrary Units (AU) The data are the results of three independent experiments. Graph bars represent standard errors (*$p < 0.05$; **$p < 0.01$).

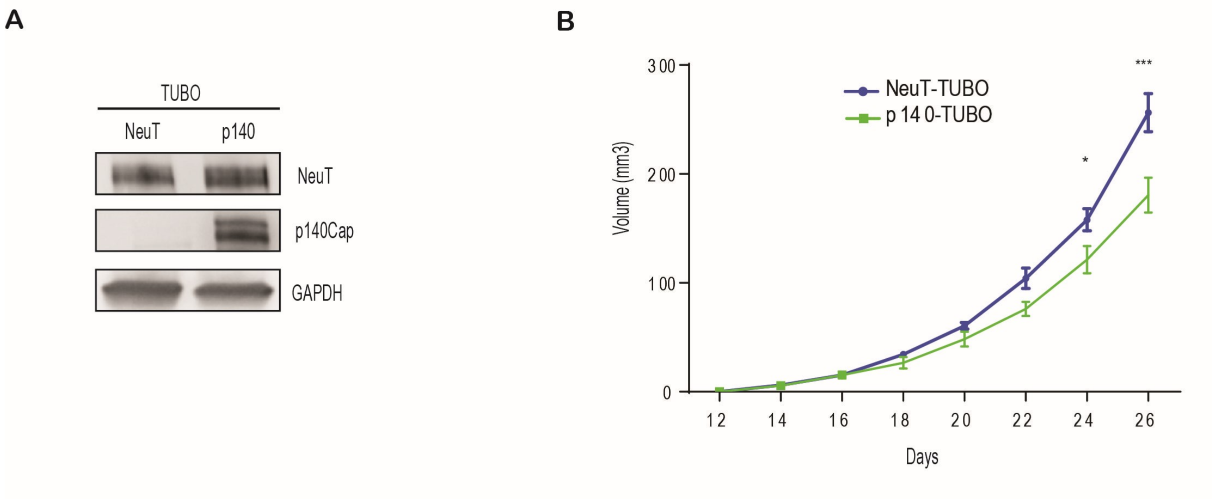

Supplementary Figure 7. Characterization of NeuT-TUBO and p140-TUBO cells

(A) NeuT-TUBO p140-TUBO cells obtained by stable infection with empty pBABE or p140Cap-pBABE retroviruses. The p140Cap over-expression was checked by western blot analysis using antibodies to NeuT, p140Cap and GAPDH, as loading control.

(B) 5 x 10^5 cells as in (A) were injected in the right fat pads of nude mice. Tumor diameters were measured every two days for 26 days. Two independent experiments were performed using 5 mice per group. Differences in tumor volume were evaluated using two-way ANOVA followed by Bonferroni multiple comparison post hoc tests (***$p < 0.001$).

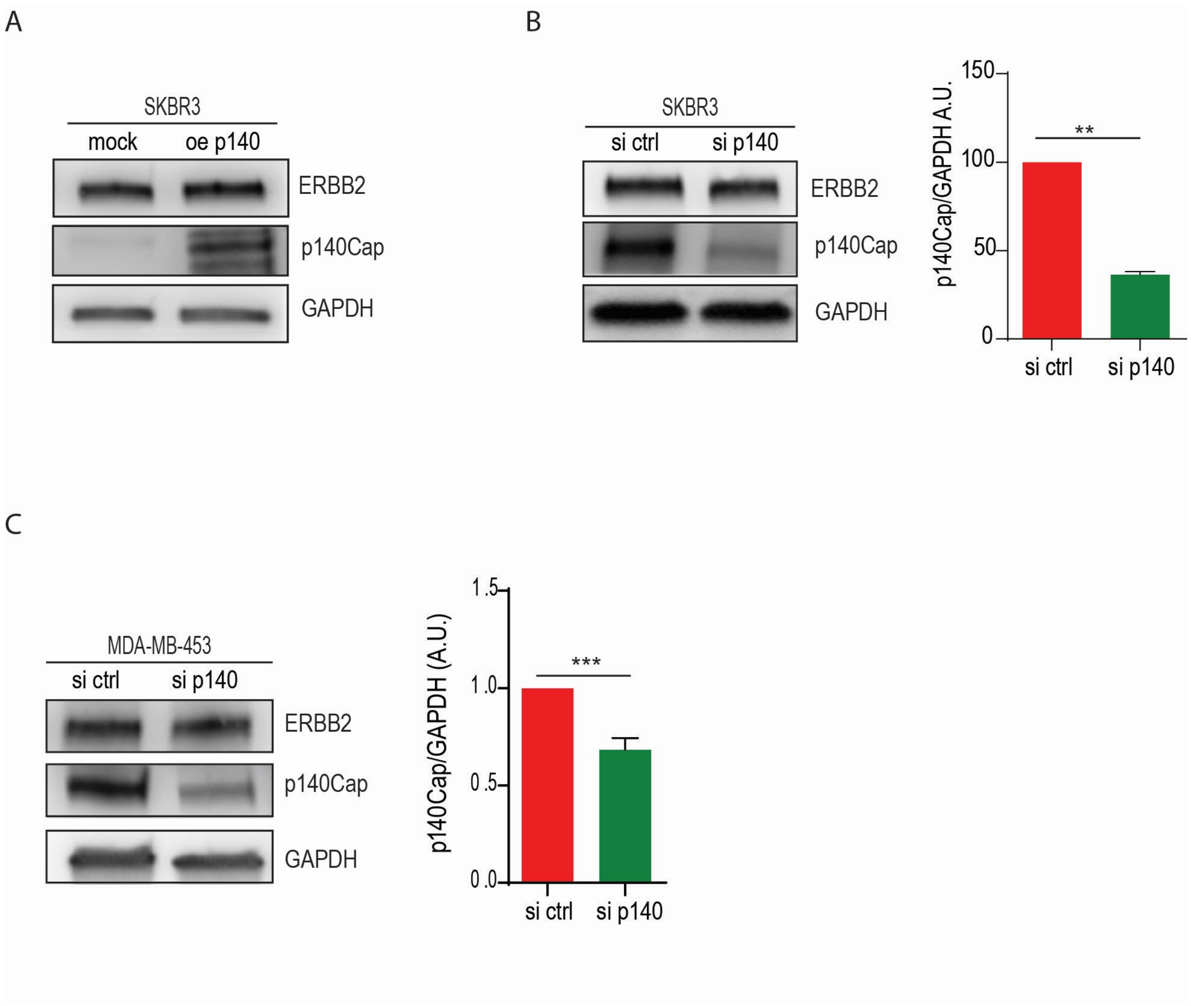

Supplementary Figure 8. Modulation of p140Cap expression in human *ERBB2* amplified SKBR3 andMDA-MB-453 cells.

(A) p140Cap over-expressing or mock SKBR3 cells obtained by stable infection with p140Cap-pBABE or empty pBABE retroviruses. The p140Cap over-expression was checked by western blot analysis using antibodies to p140Cap and GAPDH, as loading control.

(B) SKBR3 cells were transiently transfected with ON-TARGET plus human SRCIN1 small-interfering RNA (siRNA) (si p140) or ON-TARGET plus non-targeting siRNA (Dharmacon RNAi) (si ctrl). This patented approach strongly prevents off-target effects. Extracts were run on 6% SDS-PAGE to analyze p140Cap expression by western blot with antibody to p140Cap and GAPDH, as loading control.

(C) MDA-MB-453 cells were transiently transfected with ON-TARGET plus human SRCIN1 small-interfering RNA (siRNA) (si p140) or ON-TARGET plus non-targeting siRNA (Dharmacon RNAi) (si ctrl), and processed as in B.

In B and C, the histogram shows the ratio between p140Cap and GAPDH levels in arbitrary units (A.U.). Statistical significative differences were evaluated using umpaired t tests (**$p < 0.01$; ***$p < 0.001$). Error bar: SEM.

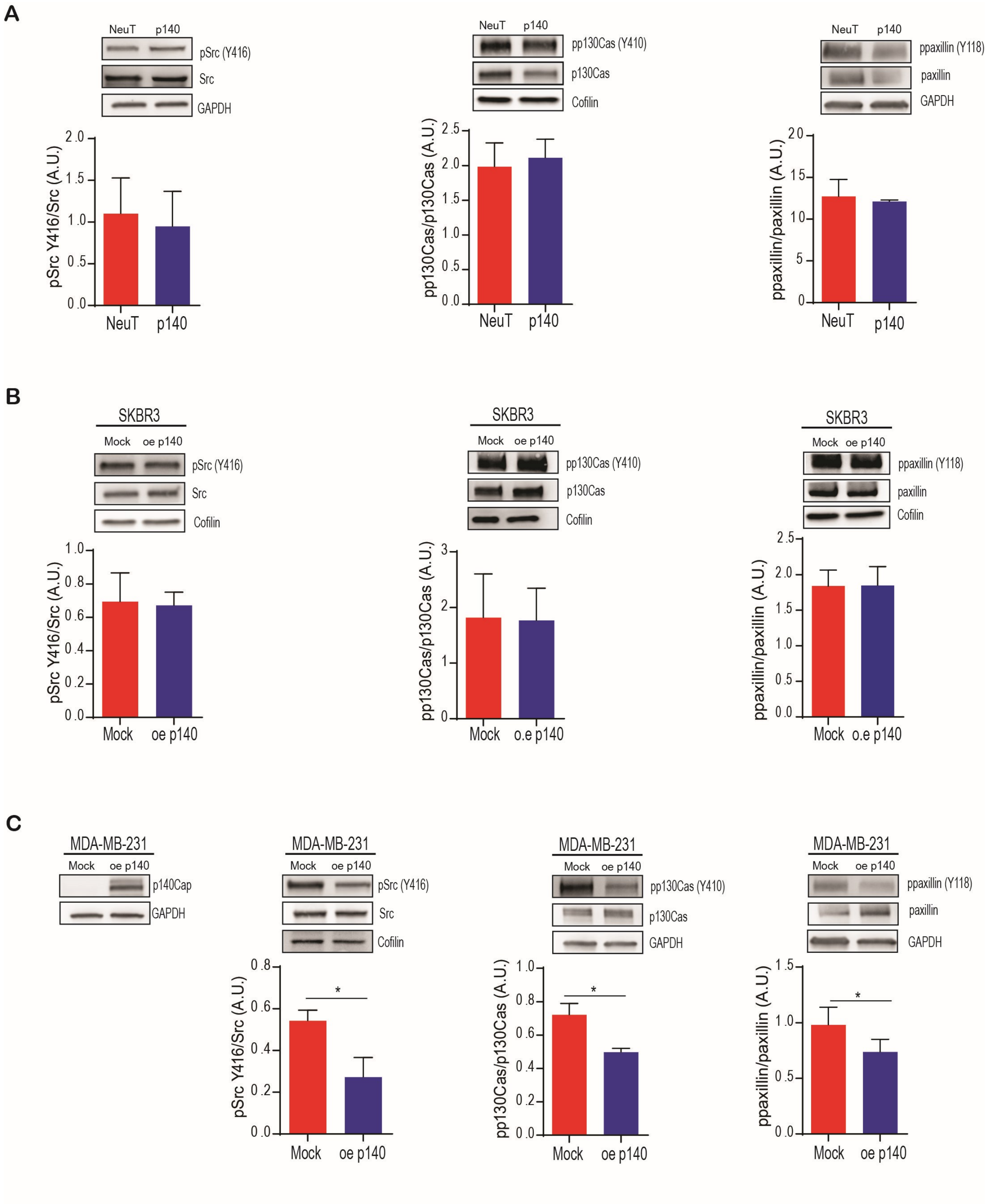

Supplementary Figure 9. p140Cap expression does not affect the activation of the Src kinase and the phosphorylation of its effectors, p130Cas and paxillin.

(A) Protein extracts from NeuT and p140 primary cancer cells were run on 6% SDS-PAGE and stained with antibodies to pSrc (Y416), Src, pp130Cas (Y410), p130Cas, pPaxillin (Y118), Paxillin and GAPDH or cofilin for loading control.

(B) Protein extracts from p140Cap over-expressing or mock SKBR3 cells were processed as in A.

(A)(C) Protein extracts from p140Cap over-expressing or mock MD-MB-231 cells were processed as in A. p140Cap antibodies were used in the left panel to assess the over-expression. Statistical significative differences were evaluated using umpaired t tests (*$p < 0.05$). Error bar: SEM.

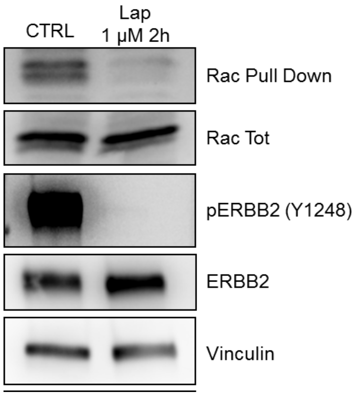

Supplementary Figure 10. Lapatinib treatment affects ERBB2 tyrosine phosphorylation and Rac activity in SKBR3 cells.

SKBR3 cells were treated with 1 micromolar Lapatinib for 2 hours. Activated Rac was detected by pull/down assay incubating 2.5 mg of cell extracts, with recombinant PAK CRIB domain bound to Glutathione Sepharose beads. Eluted material was revealed by Western blot on 12% SDS-PAGE with anti Rac antibodies (upper panel). Total amount of Rac protein from cell extracts is shown in the lower panel. The inhibition of ERBB2 activity by Lapatinib was assessed by western blot analysis using the antibody to p1248 Tyrosine on ERBB2.

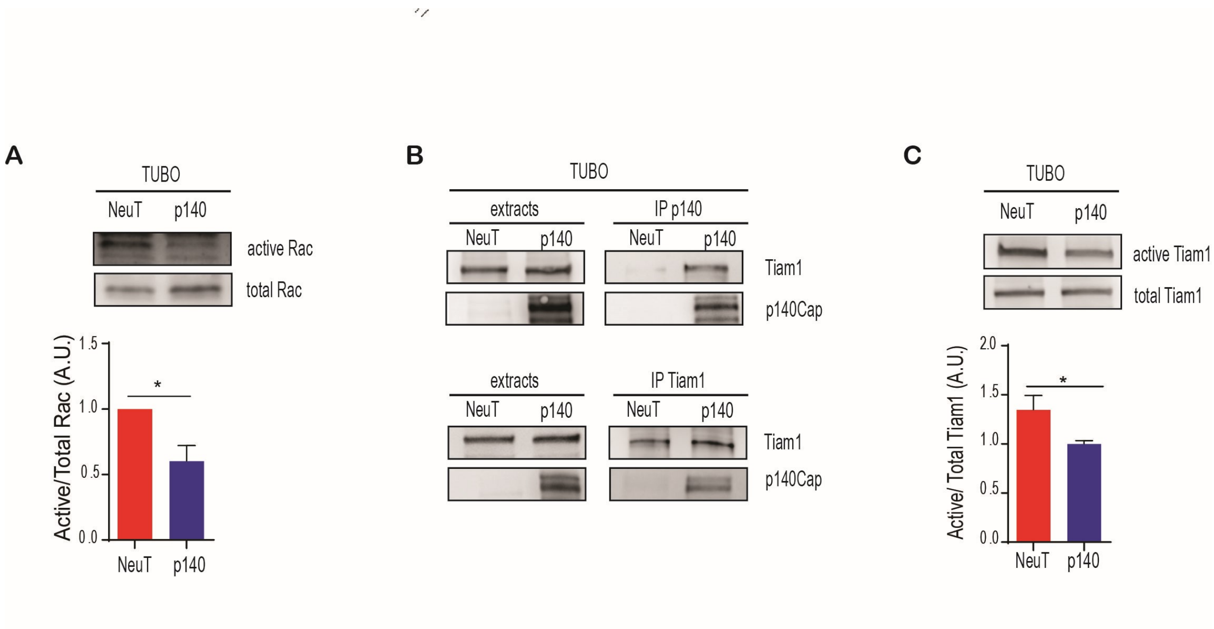

Supplementary Figure 11. Tiam1/Rac axis in NeuT-TUBO and p140-TUBO cells

(B) Active Rac pull down from NeuT-TUBO and p140-TUBO cells. Eluted material (upper panels) and cell extracts (lower panels) run on 12% SDS-PAGE revealed with anti Rac antibodies. Histograms show the ratio between active and total Rac protein levels in arbitrary units (A.U.) from four independent experiments. Statistical significative differences were evaluated using umpaired t tests (*$p < 0.05$; **$p < 0.01$). Error bar: SEM.

(C) Extracts from NeuT-TUBO and p140-TUBO cells were immunoprecipitated with antibodies to p140Cap (upper panels) or Tiam1 (lower panels). Cell extracts and immunoprecipitates were run on 6% SDS-PAGE and blotted with antibodies to p140Cap and Tiam1. Representative images from five independent experiments are shown.

(D) The level of active Tiam1 was determined using the active Rac-GEF assay kit in NeuT-TUBO and p140-TUBO cells. Equal amount of extracts were incubated for 1h at 4°C with Rac G15A agarose beads. Active Tiam1 and total Tiam1 levels were determined using an anti-Tiam1 antibody for western-blot detection, from eluted material and input fractions, respectively. The histogram represents the quantification of active Tiam1 in three independent experiments, normalizing active Tiam1 levels to the corresponding total Tiam1 levels in arbitrary units (A.***U.)***

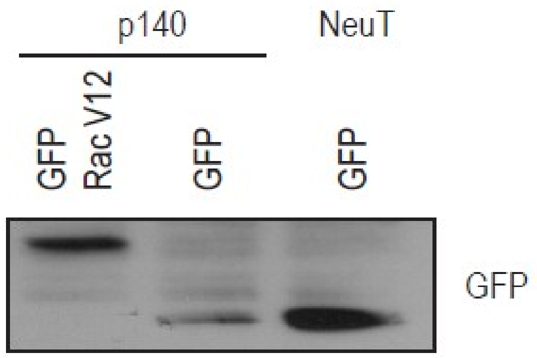

Supplementary Figure 12. GFP-RacV12 expression in p140 primary cancer cells.

Extracts from primary p140 or NeuT cancer cells were infected with retrovirus particles encoding either for GFP-RacV12 or GFP alone. Cell extracts were run on 12% SDS-PAGE, and analyzed by western blot with antibodies to GFP.

Supplementary Table 1.

**All Patient**		622
**Nodal Status**	Negative	316
	Positive	295
	x	11
	Negative	316
	1-3	205
	4+	90
	x	11
**SubType**	Luminal A	198
	Luminal B	284
	Luminal B ERBB2 Positive	65
	ERBB2 Positive	42
	Triple Negative	33
**ER**	Positive	544
	Negative	78
**PgR**	Positive	479
	Negative	143
**pT**	pT1	424
	pT2/pT3/pT4	198
**Grade**	G1	146
	G2	291
	G3	178
	N.A.	7
**Ki-67**	LOW >14%	212
	HIGH ≥14%	410
**ERBB2 Status**	Negative	515
	Positive	107
**Age**	<35	22
	35-65	364
	>65	116

Clinical and pathological information of Consecutive Cohort of breast cancer patients

The clinical and pathological information of the patients included in the TMA operated at the European Institute of Oncology (IEO) in year 2000 are reported. For some patients not all information was available. An informed consent was obtained from each subject.

Supplementary Table 2.

		p140Cap LOW	p140Cap HIGH	Pearson $\chi2$
**All Patient***		114 (22.1%)	401 (77.9%)	
**Nodal Status**	Negative	46 (17.56 %)	216 (82.44 %)	**0.0146**
	Positive	65 (26.53 %)	180 (73.47 %)	
	Negative	46 (17.56 %)	216 (82.44 %)	**<0.0001**
	1-3	35 (20.35 %)	137 (79.65 %)	
	4+	30 (41.10 %)	43 (58.90 %)	
**SubType**	Luminal A	22 (13.50 %)	141 (86.50 %)	**0.0007**
	Luminal B	53 (22.55 %)	182 (77.45 %)	
	Luminal B Her2 Positive	14 (25.93 %)	40 (74.07 %)	
	HER2 Positive	14 (36.84 %)	24 (63.16 %)	
	Triple Negative	11 (44 %)	14 (56 %)	
**ER**	Positive	88 (19.56 %)	362 (80.44 %)	**0.0002**
	Negative	26 (40 %)	39 (60 %)	
**PgR**	Positive	77 (19.35 %)	321 (80.65 %)	**0.0049**
	Negative	37 (31.62 %)	80 (68.38 %)	
**pT**	pT1	45 (12.86 %)	305 (87.14 %)	**<0.0001**
	pT2/pT3/pT4	69 (41.82 %)	96 (58.18 %)	
**Grade**	G1	11 (8.73 %)	115 (91.27 %)	**<0.0001**
	G2	54 (22.98 %)	181 (77.02 %)	
	G3	49 (32.67 %)	101 (67.33%)	
**Ki-67**	LOW	24 (13.87 %)	149 (86.13 %)	**0.0013**
	HIGH	90 (26.32 %)	252 (73.68 %)	
**Perivascular Invasion**	Absent	68 (19.94 %)	273 (80.6 %)	0.0931
	Present	46 (26.44 %)	128 (73.56 %)	
**ERBB2 Status**	Not Amplified	86 (20.33 %)	337 (79.67 %)	**0.0344**
	Amplified	28 (30.43 %)	64 (69.59 %)	
**Age**	<35	5 (25 %)	15 (75 %)	0.3094
	35-65	83 (20.65 %)	319 (79.35 %)	
	>65	26 (27.96 %)	67 (72.04 %)	

ER: estrogen receptor; PgR: progesterone receptor; pT: primary tumor size; KI67: marker of proliferation grade.

Correlation of p140Cap expression with clinic-pathological characteristics of Breast Cancer Patients. Analysis of p140Cap expression was performed on the Invasive Breast Cancer Consecutive Cohort (N = 622*) operated in the European Institute of Oncology, Milano, Italy, in the year 2000. Expression was measured by IHC on TMA. p140Cap high expression was defined

when tumors display an expression score ≥1. p-values (Pearson) were measured by Chi Square test. Note that the number of scored cases is lower than the total number of cases since: i) in some cases, individual cores detached from the slides during the manipulations; ii) clinical information was not available for all patients. In tumor tissues the IHC signals were associated with the tumor cell component and not with the adjacent or infiltrating stroma. *Only for 515 of 622 samples expression data for p140cap was available.

Supplementary Methods

Methods related to Supplementary Figure 4

Parameters: The fourth abdominal mammary glands were analyzed from at least five mice for age group. Only whole mounts that contained the entire ductal network including the primary duct and were free of mounting artifacts such as tissue folds were used for subsequent image analysis. A digital photomicrograph was taken of each whole mount using an Leika MZ6 stereo microscope fitted with an Nikon Coolpix color digital microscope camera. Within each age group, a consistent magnification was established that allowed the entire epithelial complex to be captured in a single image. For each age group, the photomicrographic settings remained constant. Four different measurements were obtained from each whole-mount image using Photoshop software. TEB count was performed only on 6 weeks of age glands.

Ductal length (pixels) was measured by drawing and measuring a straight line caliper from the most distal point of the ductal network to the nipple.

Ductal network area (pixels) was measured from a best-fit polygon that was drawn around each ductal network using the computer mouse. Each polygon was outlined without indentations so as to ensure consistency across samples.

Epithelial area (pixels), which excluded the intervening adipose tissue, was measured after the epithelial structures were outlined automatically using magic wand tool.

Epithelial density was calculated by dividing the epithelial area by ductal area.

TEB incidence in growing glands (6 weeks of age) was determined by manually tagging individual TEB with a computer-generated marker. End buds were identified as large bulbous profiles located at the termini of ducts and fitted the criteria of having a cross sectional diameter that was approximately twice that of the subtending duct.

Methods related to Supplementary Figure 5

For immunohistochemistry, slides were stained with rat monoclonal anti-CD31 (550274, BD Pharmingen) mixed with rat monoclonal anti-CD105 (550546, BD Pharmingen), rabbit polyclonal anti-Keratin 5 (PRB-160P, Covance) and guinea pig polyclonal anti-Keratins 8/18 (GP11, Progen), followed by the appropriate secondary antibodies (Jackson ImmunoResearch). Immunoreactive antigens were detected using alkaline phosphatase conjugated streptavidin (Thermoscientific) and vulcan fast red chromogen (Biocare Medical), or streptavidin peroxidase (Thermoscientific) and the DAB Chromogen System (Dako). The vascularization was analyzed evaluating CD31-105+ endothelial cells on digital images of 3 tumors per group (6 X 200 microscopic fields per sample) with Adobe Photoshop by selecting red stained vessels with the Magic Wand Tool and reporting the number of pixels indicated in the histogram window.
